# Oxidative Stress, Cytotoxic and Inflammatory Effects of Azoles Combinatorial Mixtures in Sertoli TM4 Cells

**DOI:** 10.3390/antiox12061142

**Published:** 2023-05-24

**Authors:** Sabrina Petricca, Veronica Carnicelli, Carla Luzi, Benedetta Cinque, Giuseppe Celenza, Roberto Iorio

**Affiliations:** 1 Department of Biotechnological and Applied Clinical Sciences, University of L’Aquila, Via Vetoio, 67100 L’Aquila, Italy; sabrina.petricca@univaq.it (S.P.); veronica.carnicelli@univaq.it (V.C.); carla.luzi@univaq.it (C.L.); giuseppe.celenza@univaq.it (G.C.); 2 Department of Life, Health and Environmental Sciences, University of L’Aquila, Via Vetoio, 67100 L’Aquila, Italy; benedetta.cinque@univaq.it

**Keywords:** tebuconazole, econazole, Sertoli TM4 cells, synergistic effects, oxidative stress, apoptosis, SOD, GSH, TNF-α, COX-2

## Abstract

Triazole and imidazole fungicides are an emerging class of contaminants with an increasing and ubiquitous presence in the environment. In mammals, their reproductive toxicity has been reported. Concerning male reproduction, a combinatorial activity of tebuconazole (TEB; triazole fungicide) and econazole (ECO; imidazole compound) in inducing mitochondrial impairment, energy depletion, cell cycle arrest, and the sequential activation of autophagy and apoptosis in Sertoli TM4 cells (SCs) has recently been demonstrated. Given the strict relationship between mitochondrial activity and reactive oxygen species (ROS), and the causative role of oxidative stress (OS) in male reproductive dysfunction, the individual and combined potential of TEB and ECO in inducing redox status alterations and OS was investigated. Furthermore, considering the impact of cyclooxygenase (COX)-2 and tumor necrosis factor-alpha (TNF-α) in modulating male fertility, protein expression levels were assessed. In the present study, we demonstrate that azoles-induced cytotoxicity is associated with a significant increase in ROS production, a drastic reduction in superoxide dismutase (SOD) and GSH-S-transferase activity levels, and a marked increase in the levels of oxidized (GSSG) glutathione. Exposure to azoles also induced COX-2 expression and increased TNF-α production. Furthermore, pre-treatment with N-acetylcysteine (NAC) mitigates ROS accumulation, attenuates COX-2 expression and TNF-α production, and rescues SCs from azole-induced apoptosis, suggesting a ROS-dependent molecular mechanism underlying the azole-induced cytotoxicity.

## 1. Introduction

Recent decades have seen an emergence regarding the harmful effects of environmental and lifestyle stressors on human health. In particular, infertility is emerging as a worldwide health problem; the percentage of men affected by reproductive impairments grows by about 0.29% per year, according to the “Global Burden of Disease Study” [1,2]. Many environmental toxicants have been shown to elicit hormone-mimetic activities and endocrine disruption effects, resulting in male fertility impairment and alterations in reproductive functions [3,4], such as trans-generational adverse effects, with genetic and molecular implications involving semen quality and/or the spermatogenesis process [5,6]. 

In the last few decades, there has been increasing awareness about azole compounds, a class of anti-fungal chemicals, currently used as pesticides and as active principles in therapeutic and personal care products [7]. Triazole and imidazole fungicides can be found as contaminants in many environmental matrices, such as surface water and sediment, soil, as well as in agricultural products, due to their physical and chemical characteristics, of which low biodegradability and volatility and high physico-chemical persistence are noted [7,8]. Azoles act by inhibiting the biosynthesis of ergosterol, the main membrane constituent of fungal cells, directly blocking lanosterol-14α-demethylase (CYP51) [9]. Therefore, due to their mechanism of action, azoles can interact with mammalian enzymes belonging to the cytochrome P450 family (CYPs), thus interfering with key enzymes responsible for steroidogenesis (e.g., aromatase CYP19). Effects on reproductive development by different azole compounds, alone or in mixtures, have been reported [10,11,12,13,14,15,16]. 

The pathophysiology of reproductive dysfunctions is difficult to elucidate due to a plethora of interlinked endogenous pathways. Oxidative stress (OS) is currently considered one of the major underlying events which contribute to the etiopathology of a wide variety of health issues, including reproductive disorders, as reported in both animal and human models [17,18]. The endogenous antioxidant pattern (enzymatic and not enzymatic) is responsible for guaranteeing the proper redox balance in all biological systems. When an excessive accumulation of ROS is not scavenged because the antioxidant defense is lacking, OS occurs. Interestingly, evidence of ROS accumulation by triazole and imidazole derivates, besides their capacity in interfering with the steroid biosynthesis, has been shown in several biological models [19]. Furthermore, the induction of OS and lipid peroxidation in many cell types [20,21,22,23] related to possible azole-induced toxicity [24,25,26,27] has been described. In particular, a link between ROS generation and different types of cell death has been identified [28]. Additionally, alterations in the antioxidant pattern have been reported, as well as in the glutathione content and enzyme specific activities in kidney-proximal epithelial rat cells [29]. Finally, human cell models exposed to ketoconazole have been shown to undergo OS, mitochondrial toxicity, and apoptosis [30,31]. 

In this scenario, the concern for studying the effects of toxicant-mixtures, rather than single compounds, to outline a more complete environmental risk assessment, is currently growing [32].

In this regard, our research group have recently demonstrated that different azole compounds (e.g., ketoconazole, miconazole, and prochloraz), tested alone and in mixtures, have the ability to induce OS, mitochondrial dysfunctions, and cell death in TM4 mouse Sertoli cells (SCs) [33].

SCs are considered to be one of the main targets of environmental toxicants in male reproductive dysfunctions [15,16]. SCs have a key role in maintaining functional spermatogenesis, providing nutritional and structural support for germ cells. They are also essential for the testis because of their production of follicle-stimulating hormone receptor (FSHR) and the androgen-binding protein (ABP) [34], and crucial for their direct implication in the blood–testis barrier (BTB) and in the release of various immunomodulatory factors [35]. In this context, a high level of proinflammatory cytokines produced by SCs, especially tumor necrosis factor-alpha (TNF-α), affects BTB functions, thereby leading to the impairment of spermatogenesis [36,37,38,39,40]. In performing their task, SCs also regulate and maintain the appropriate cellular redox status of sperm cells that exhibit a high ROS accumulation. 

OS has been shown to be closely related to inflammation [41] and, consequently, to initiate a potential vicious cycle, eventually leading to an exacerbation of tissue damage and, finally, to a negative cell fate, with apoptotic or even autophagic death. As a result, a disruption to the physiological reproductive functions may occur that may result in a variety of reproductive disorders, or even subfertility/infertility conditions [42]. In this context, the potential impact of cyclooxygenase (COX)-2 in the regulation of testicular function and male fertility has also been reported [43,44,45]. Currently, it is well known that elevated ROS levels have been shown to have a key role in inducing apoptosis [46]. Additionally, a strong link between mitochondrial activity and ROS has been reported [46,47,48,49].

Recently, the synergistic activity of tebuconazole (TEB) and econazole (ECO) in inducing mitochondrial dysfunctions, energy imbalance, and the sequential activation of autophagy and apoptosis in TM4 SCs was ascertained by our research group [50]. This may also be of concern considering the large clinical use of ECO (topical, oral, and parenteral) in treating dermatomycoses.

Therefore, in the light of these emerging concerns, the aim of the present work was to evaluate whether the individual and combined potential of TEB and ECO in inducing in vitro toxicity in TM4 SCs arises from early alterations in the redox status, and is thus directly related to the induction of OS. To this end, an assessment of changes in intracellular ROS production, the detection of the glutathione (GSH) pool content, and analyses of the enzymatic antioxidant defense (catalase, superoxide dismutase, GSH reductase, GSH-S-transferase, and GSH peroxidase) were performed. The potential implication of ROS to azoles-induced cytotoxicity was investigated through pre-treatment with the commonly used ROS-inhibitor N-acetylcysteine (NAC). Finally, given the impact of TNF-α and COX-2 in modulating SCs physiology and the testis environment, their protein expression levels were also assessed.

## 2. Materials and Methods

### 2.1. Cell Culture and Treatments

The Sertoli TM4 cells (ATCC^®^ CRL1715™) purchased from the American Type Culture Collection were routinely maintained in a humidified atmosphere with 5% CO_2_ at 37 °C and seeded at 1 × 10^4^ cells/cm^2^ density. They were cultured in Dulbecco’s modified Eagle medium/ Ham’s F-12 (1:1) Mix (Corning Life Sciences, Manassas, VA, USA), containing 15 mM 4-(2-hydroxyethyl)-1-piperazineethanesulfonic acid (HEPES), 2 mM L-glutamine, and sodium bicarbonate, complemented with heat-inactivated 5% horse serum (HS) and 2.5% fetal bovine serum (FBS) (EuroClone, Pero, MI, Italy), and with penicillin 100 IU/mL and streptomycin 100 µg/mL (Corning, Manassas, VA, USA). Cells were routinely cultured and detached until they reached about 80% confluence in the described standard conditions. Cell proliferation and viability were determined using the Trypan-blue exclusion assay. For the experiments with azole compounds, 24 h after seeding cells were then exposed to 40 µM TEB, 20 µM ECO (≥98% purity, from European Pharmacopoeia, Council of Europe, Strasbourg, France), and their mixture (MIX) at the times and the tested concentrations indicated below. Azoles were solubilized in dimethyl sulfoxide (DMSO), and stock solutions were prepared to maintain the same final concentration of 0.04% DMSO in single and combination experiments. We excluded any significant differences between control groups exposed or not exposed to 0.04% DMSO, in cell viability, cytotoxicity, and ROS experiments. Therefore, analyses were performed comparing azole-treated with vehicle-control groups (CTR). For NAC experiments, cells were pre-treated for 1 h with 1 mM NAC and incubated for the specific times of the experiments indicated below.

### 2.2. Intracellular Reactive Oxygen Species (ROS) Detection

The detection of intracellular ROS generation was analyzed by using 2′,7′-dichlorofluorescein diacetate (DCFH-DA) (Molecular Probes, Eugene, OR, USA), as previously described [51,52]. In brief, immediately after treatments, samples were incubated at 37 °C for 30 min with 1 µM DCFH-DA. Cells exposed to each treatment (or not) were subsequently collected, washed twice in ice-cold phosphate-buffered saline (PBS; pH 7.4), and then analyzed using a spectrofluorometer (Perkin-Elmer LS-50B; Waltham, MA, USA) at the excitation wavelength of 502 nm and 524 nm for the emission, in order to detect the ROS production. To obtain a positive control for the ROS production, cells were exposed for 1.5 h to 500 µM tert-butyl hydroperoxide (t-BHP). 

### 2.3. Enzymatic Activity Assays

Activities of the antioxidant enzymes were assayed using spectrophotometric methods, as previously reported [33,53]. Briefly, after 3 h of incubation with azoles, cells were washed in ice-cold PBS and buffered in a solution containing 1% (*v*/*v*) Triton X-100, 1 mM ethylene diamine tetra-acetic acid (EDTA) and 50 mM Tris–HCl at pH 7.4. After disruption with several freeze–thawing cycles, the supernatants were collected (17,000 g, for 10 min, at 4 °C), and after protein quantification they were subsequently analyzed. Total glutathione peroxidase (GPx) activity, considered as se-dependent and se-independent, and the glutathione reductase (GR) activity of each protein extract, were evaluated with a spectrophotometer (Perkin-Elmer Lambda19; Waltham, MA, USA), monitoring the oxidation of nicotinamide adenine dinucleotide phosphate (NADPH) to a wavelength of 340 nm, at 25 °C. The reaction mixtures contained 50 mM potassium phosphate buffer at pH 7.0, 1 mM EDTA, 0.2 mM NADPH, 70 µM t-BHP, 0.01 U/mL GSH reductase and 1 mM GSH or 1 mM GSSG, respectively, and the adequate amount of the protein extract. The glutathione transferase (GST) activity was assayed at 340 nm by determining the conjugation rate of GSH to 1-chloro-2,4-dinitrobenzene, with a reaction mix containing 0.1 M potassium phosphate buffer at pH 6.5, 1 mM 1-chloro-2,4-dinitrobenzene, 2 mM GSH, and the proper volume of the supernatants. To assay catalase (CAT) activity, its 10 mM decomposition at a wavelength of 240 nm was measured. One unit was defined as 1 µmol of H_2_O_2_ reduced/min at 25 °C. The superoxide dismutase (SOD) activity was determined with the colorimetric method (Thermo Fisher Scientific; Life Technologies Corp, Carlsbad, CA, USA). The assay was performed as the manufacturer’s instructions indicated; in brief, all types of SOD (e.g., Mn, Cu/Zn, and FeSOD) activity were measured for each sample in a 96-well microplate. After resuspending the cells in the diluent, a colorless substrate was added and the mixture was then incubated at room temperature with Xanthine Oxidase Reagent for 20 min. The superoxide produced, in the presence of oxygen, converted the substrate into a yellow product, detected at a wavelength of 450 nm. A less yellow product reveals SOD increasing levels. The enzymes specific activities were expressed as U/mg protein, where U is considered in nmol/min.

### 2.4. Detection of GSH and GSSG Intracellular Content

The intracellular contents of GSH and GSSG were detected with a colorimetric assay (Thermo Fisher Scientific; Life Technologies Corp, Carlsbad, CA, USA). After 3 h of incubation with azoles, the analyses were performed on cell lysates in line with the manufacturer’s instruction; in brief, cell lysates were added into a 96-well microplate (Corning Inc., New York, NY, USA), earlier prepared in an ice-cold 5% solution of 5-sulfo-salicylic acid dehydrate (Sigma-Aldrich, St. Louis, MO, USA), and subsequently diluted in the supplied buffer. Once the specific substrate had been added, the reaction started producing a colored product whose absorbance was detected at 405 nm by the Infinite M Plex, multimode (TECAN, Salzburg, Austria, Europe). The analyses were replicated at least three times per assay. 2-vinylpyridine (2-VP) (Sigma-Aldrich, St. Louis, MO, USA) was used to block free thiols and GSH, when the content of GSSG (the oxidized form of GSH) was analyzed; the GSSG content was then determined by comparing a 2-VP standard curve.

### 2.5. TNF-α Detection by ELISA

To perform TNF-α detection, TM4 cells were seeded (1 × 10^5^ cells) in a 24-well plate (Corning Inc., New York, NY, USA) and incubated for 24 h at 37 °C and 5% CO_2_. Afterwards, cells were treated with TEB, ECO, and MIX for 6 h of exposure. At the end of the treatments, the supernatants were collected and centrifuged in order to determine the TNF-α concentration using the ELISA MAX Deluxe Set (from BioLegend Inc., San Diego, CA, USA) according to the manufacturer’s protocol. After stopping the reaction, positive wells turned from blue into a yellow-colored product, and subsequently the absorbance was read at 450 nm with a Nunc Plate Maxisorp reader (Infinite M Plex, multimode; TECAN, Salzburg, Austria, Europe). Data are expressed as pg/mL.

### 2.6. Western Blotting

Total proteins extracts from control and treated cells after 6 h of incubation with azoles, in the presence or absence of NAC, were obtained by using a lysis buffer containing 10 mM HEPES at pH 7.2, 5 mM MgCl_2_, 142 mM KCl, 1 mM EDTA, 1 mM phenyl methyl-sulfonyl fluoride (PMSF), and a cocktail of protease inhibitors (Sigma-Aldrich, St. Louis, MO, USA). Extracts were run in a 10% sodium dodecyl-sulfate polyacrylamide gel electrophoresis (SDS-PAGE) and transferred onto polyvinylidene fluoride (PVDF) membranes; 5% non-fat dry milk was diluted in Tris-buffered saline with 0.1% Tween-20 (TBST), and used as blocking solution O/N at 4 °C. After blocking incubation, filters of PVDF were maintained at room temperature for 2 h with the anti-COX-2 primary antibody (Cell Signaling, Danvers, MA, USA) diluted 1:1000 in 1% non-fat dry milk in TBST. After washing, PVDF were incubated at room temperature for 1 h with the corresponding horseradish peroxidase (HRP)-conjugated secondary antibody (Immunological Sciences, Rome, Italy), diluted 1:5000 in TBST 1% non-fat dry milk. ECL West Pico Plus chemiluminescent solution was used to reveal the signal with a ChemiDoc XRSplus imaging system (Bio-Rad Laboratories, Hercules, CA, USA), and the optical densities of blot protein bands were analyzed with ImageJ software (US National Institutes of Health, Bethesda, MD, USA). Values represent the protein expression levels (arbitrary units, a.u.), with ß-actin used as the loading control.

### 2.7. Apoptosis Detection with Flow Cytometry Analyses

A PE Annexin V dye, supplied in the Apoptosis Detection Kit I from BD Biosciences (San Jose, CA, USA), in conjunction with a vital dye such as 7-Amino-Actinomycin (7-AAD), was used to detect apoptotic cells according to the manufacturer’s instructions. Briefly, after treatments, the cells were collected, twice washed with cold PBS, and resuspended in the buffer containing 0.1 M HEPES/NaOH (pH 7.4), 1.4 M NaCl, and 25 mM CaCl_2_ at a concentration of 1 × 10^6^ cells/mL. The reagents were pre-diluted for use at the recommended volume per test. After staining with PE Annexin V and 7-AAD in the dark, at room temperature for the recommended times, samples were then washed and subsequently analyzed using a flow cytometer. Data from 10,000 events per sample were collected and analyzed using FACSCalibur flow cytometry (BD Instruments Inc., San José, CA, USA) equipped by Cell Quest software (BD Instruments Inc.) to calculate the percentages of apoptotic and necrotic cells. 

### 2.8. Statistical Analyses

All statistical analyses were performed in at least three independent experiments. Unless otherwise indicated, data in this study are reported as the mean ± standard error (SE). Sigma Stat 2.03 software (SPSS, Chicago, IL, USA) was used to extrapolate the statistical significance of differences between group means. To analyze the comparisons among multiple groups, an ANOVA test followed by Holm–Sidak or Dunnett’s Method was used. ANOVA on ranks (Kruskal–Wallis test) followed by the Tukey–Kramer test were used for analyses of CAT and GR/(GPx + GST) ratio. For NAC experiments, values were determined by ratios between exposed (X_NAC_) and not exposed to NAC (X) groups ([X_NAC_−X/X]) in azole and CTR samples, and expressed as the media ± SE. A value of *p* < 0.05 versus CTR was considered statistically significant.

## 3. Results

### 3.1. Azoles Induce an Early and Sustained Increment of ROS in TM4 Sertoli Cells

In our recent study, TEB (40 µM) and ECO (20 µM) ability, alone or in combination to induce mitochondrial membrane potential (ΔΨm) loss were shown starting from 3 h of exposure [50]. In order to evaluate whether the azoles-induced mitochondrial depolarization was associated with alterations in the redox status, the trend of ROS formation over time (from 0.5 h up to 24 h) by detecting the DCFH-DA fluorescent signal in azole-exposed and not exposed SCs was examined.

**Figure 1:**
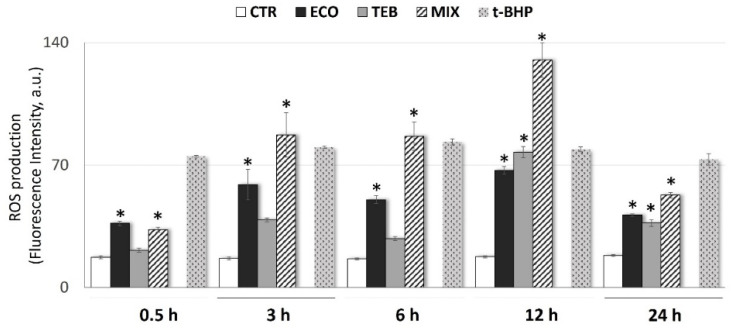
**Exposure to azole fungicides induces early and sustained ROS production over time in TM4 cells.** The graph represents the over-production of ROS in SCs not exposed (CTR) or exposed to azole compounds ECO and TEB, alone and in mixture (MIX), for an extended time-course from 30 min up to 24 h; intracellular ROS levels were measured with a fluorimeter detecting DCFH-DA dye fluorescence intensity (arbitrary units, a.u.). To ensure a positive control, cells were incubated for 1.5 h with 500 µM tert-butyl hydroperoxide (t-BHP). Values are considered as the media ± SE, from three independent experiments; OneWay ANOVA, followed by Dunnett’s test; * *p* < 0.05 vs. CTR.

As shown in Figure 1, immediately after 30 min of exposure ECO and MIX elicited a significant ROS increase that was progressive and sustained over time for up to 24 h. Similarly, TEB treatment also induced ROS generation, although significant values were recorded starting from 12 h of exposure.

In particular, the main peaks of ROS production were deputed to the MIX (~1.9-fold increase at 0.5 h; ~5.2-fold increase at 3 h; ~5.3-fold increase at 6 h, ~7.4-fold increase at 12 h; ~2.9-fold increase at 24 h), reinforcing the previously revealed synergistic action of the examined binary combination. A significant but lesser increase in ROS was revealed for ECO alone (from a minimum of ~2.1-fold increase up to a maximum of ~3.8-fold increase) with respect to the CTR. Overall, these findings confirm that azole compounds, alone and in combination, are able to induce different time-dependent degrees of ROS production in TM4 cells (TEB < ECO < MIX). This is also in line with our previous results describing the decrease in ΔΨm and alterations in the mitochondrial morphology in SCs after exposure to azole compounds.

### 3.2. Azoles Alone and Mixtures Impair the Antioxidant Enzyme Pattern, Especially in SOD Activity Levels

To investigate changes in the cellular redox status induced by azoles more deeply, antioxidant enzymatic pattern activities were assayed. 

**Figure 2:**
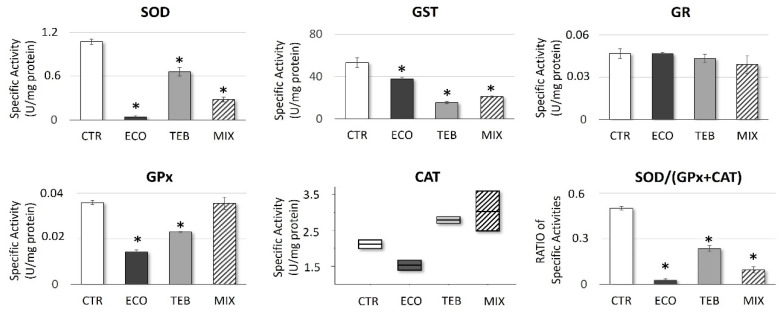
**Imbalance of enzymatic antioxidant activities in azole-exposed TM4 cells.** Azole-treatments significantly affected SOD activity levels, causing an altered SOD/(GPx + CAT) ratio. The specific activities of the tested enzymes were analyzed in samples exposed or not exposed (CTR) to azoles after 3 h of incubation. Values expressed as specific activities or ratios, as indicated, represent the media ± SE from three independent experiments; OneWay ANOVA followed by Dunnett’s test or Holm–Sidak methods; ANOVA on ranks (Kruskal–Wallis test followed by Tukey Test) (CAT activity); * *p* < 0.05 vs. CTR.

At 3 h of exposure, ECO, TEB, and MIX impaired the specific activities of the antioxidant enzymes in SCs, as shown in Figure 2. Specifically, a marked decrease in the SOD-specific activity levels was observed, with a greater impact on ECO- and MIX-treated cells. CAT specific activity analyses showed variations for all treatments with respect to the recorded CTR levels, albeit not significantly. Additionally, the specific activity of GPx was shown to undergo azoles-induced alterations, assuming a significant and negative trend in ECO and TEB samples. 

In this context, a marked drop in SOD specific activity is a well-defined symptom of a general impairment in the antioxidant enzymes’ efficiency. To further confirm, a significant reduction in the SOD/(GPx + CAT) ratio was recorded (CTR = 0.5 ± 0.11; ECO = 0.026 ± 0.01; TEB = 0.235 ± 0.018; MIX = 0.095 ± 0.019), suggesting an affected ability for superoxide anion (O_2_^−^) scavenging. Moreover, confirming the azole-dependent pro-oxidative picture already highlighted, a significant reduction in the GST specific activity was also detected in all treatment conditions (CTR = 53.01 ± 4.57; ECO = 37.71 ± 1.39; TEB = 15.29 ± 1.06; MIX = 20.89 ± 1.03).

### 3.3. Azole-Induced Alteration in the GSH/GSSG Ratio Reflects an Increase in GSSG Intracellular Content

**Figure 3:**
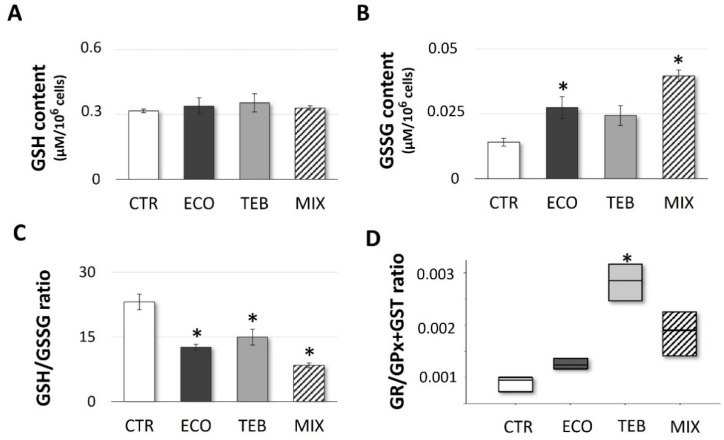
**Azoles’ exposure results in significant alterations of GSH homeostasis.** Levels of (**A**) reduced (GSH), (**B**) oxidized glutathione (GSSG), (**C**) GSH/GSSG, and (**D**) GR/(GPx + GST) ratios in CTR and azoles-treated groups after 3 h of incubation. Values from three independent experiments are expressed as the media ± SE; OneWay ANOVA, followed by Dunnett’s test; ANOVA on ranks (Kruskal–Wallis test followed by Tukey Test) (GR/(GPx + GST) ratio); * *p* < 0.05 vs. CTR.

To further investigate OS effects induced by azoles, parallel to monitoring the antioxidant enzymatic activities, we measured the total GSH content and its oxidized GSSG form at 3 h of exposure. As shown in Figure 3, although no significant variations have been recorded for intracellular GSH content (Figure 3A), azoles induced a significant increase in the GSSG levels (Figure 3B), thereby leading to alterations in the GSH/GSSG ratio (Figure 3C), more incisively expressed by the MIX treatment (CTR = 23.11 ± 1.82; ECO = 12.63 ± 0.66; TEB = 14.96 ± 1.86; MIX = 8.39 ± 0.55). In this context, the ability to maintain the GSH reservoir may reflect the negative trend observed in GSH-utilizing enzymes, including GST and GPx, rather than a better efficiency in GSH recycling. Therefore, an increment of the GR/(GPx + GST) ratio was also recorded (Figure 3D). Consistent with ROS generation, in this case TEB also exhibited a minor impact in terms of OS.

### 3.4. Azole Exposure Affects the SCs Inflammatory State, Inducing TNF-α and COX-2 Expression

**Figure 4:**
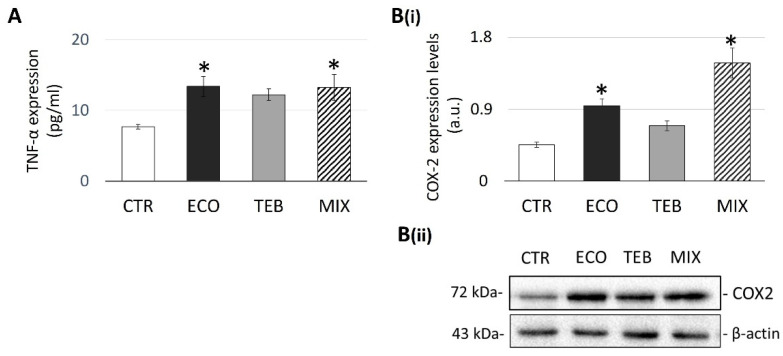
**Differential up-regulation of TNF-α and COX-2 expression by azoles in TM4 cells.** Protein expression levels of TNF-α (**A**) and COX-2 (**B(i)**) in TM4 treated cells at 6 h of incubation, compared with the CTR. (**B(ii)**) Representative images of COX-2 blot band in CTR and treated samples with azoles at 6 h of incubation. Values from three independent experiments are expressed as the media ± SE; OneWay ANOVA followed by Dunnett’s test or Holm–Sidak methods; * *p* < 0.05 vs. CTR.

OS and inflammatory responses are interdependent processes, strictly related to male subfertility/infertility conditions. Therefore, to investigate whether azole-induced OS in SCs was associated with the production/release of inflammatory mediators, we analyzed TNF-α and COX-2 expression levels, considered as principal markers of inflammation. As shown in Figure 4A, TNF-α production at 6 h of exposure was positively modulated in all the treated samples, although no remarkable differences were observed when comparing TEB-exposed cells with CTR (CTR = 7.687 ± 0.31; ECO = 13.377 ± 1.42; TEB = 12.207 ± 0.85; MIX = 13.21 ± 1.83). These results were further confirmed by the analysis of COX-2 expression levels, which correlated with significant values for ECO- and MIX-treated samples, specifically to a higher extent for the MIX treatment (Figure 4B). 

### 3.5. NAC-Improved Cell Survival in OS Is Associated with Mitigation in ROS Production, Apoptosis, and TNF-α and COX-2 Levels

**Figure 5:**
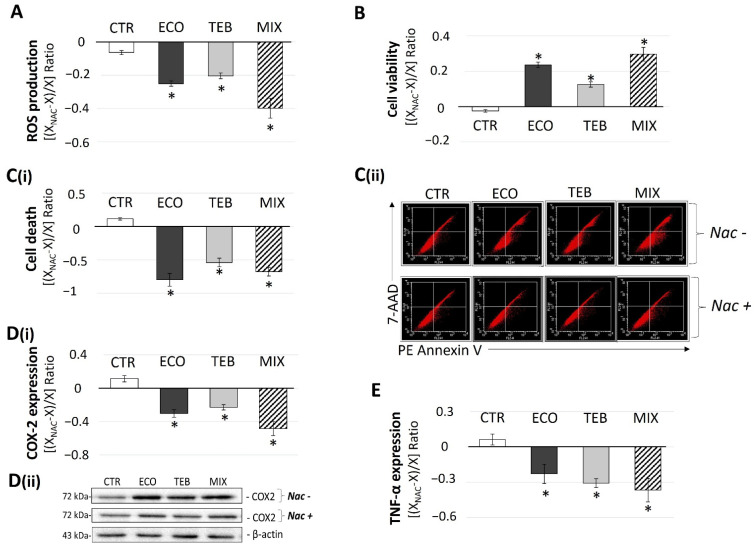
**Anti-oxidative and cytoprotective effects of NAC in TM4 cells exposed to azoles.** NAC treatment partially rescues azole-induced cytotoxicity in TM4 cells by decreasing ROS production (3 h; (**A**)), improving cell viability (48 h; (**B**,**C(i)**)), and attenuating inflammatory mediators’ expression (6 h; (**D(i)**,**E**)). (**C(ii)**) Representative images of FACS profiles of samples not exposed (CTR) or exposed to azoles for 48 h and stained with annexin V and 7-AAD, in the presence (+) or in the absence (−) of NAC. (**D(ii)**) Representative images of COX-2 blot band in CTR and treated samples with the tested antifungal compounds at 6 h of exposure, in the presence (+) or in the absence (−) of NAC. Values from three independent experiments are determined by ratios between exposed (X_NAC_) and not exposed to NAC (X) groups ([X_NAC_ − X/X]) in azole and CTR samples, and expressed as the media ± SE; OneWay ANOVA followed by Dunnett’s test; * *p* < 0.05 vs. CTR.

Our results indicated that OS could be a key route in inducing the azole-dependent cytotoxicity of SCs. Therefore, we checked the hypothesis that the concurrency of ROS may potentially contribute to the onset of azole-induced toxicity, by using a pre-treatment with the commonly used ROS-inhibitor and intracellular GSH precursor NAC. ROS generation analyses in the presence of NAC demonstrated an expected significant reduction in the treated samples (Figure 5A). In addition, to investigate the potential implication of ROS to azoles-induced cell death, cellular survival and the percentage of cell death in azole-treated samples were assessed at 48 h and exposed or not exposed to NAC, respectively, with Trypan-blue assay and FACS analyses. The results showed a significant increase in cell survival (Figure 5B), confirmed by Annexin-V analyses, that showed an equally significant attenuation of the azole-induced cell death (Figure 5C), thus demonstrating a direct contribution of ROS to the azole-induced cytotoxicity. Finally, to explore ROS-related inflammatory effects, we analyzed the expression levels of both markers, TNF-α and COX-2, at the previously tested times in the presence of NAC. As shown in Figure 5, the levels of TNF-α (Figure 5E) and the COX-2 expression (Figure 5D) were significantly reduced in azole-treated cells.

## 4. Discussion

As pivotal elements for an adequate development of testis and the spermatogenesis process, SCs are a commonly used cellular model for testing cytotoxicity and its relationship with male reproductive system dysfunctions, since they have been ascertained to be the cellular target of many environmental and chemical toxicants [54]. As a consequence, many conditions of male subfertility or infertility might be due to SCs impairment [55,56,57,58,59,60,61], potentially subsisting due to exposure to a plethora of xenobiotics and chemical mixtures in the daily living environment [62,63]. Nowadays, emerging toxicant-mediated mechanisms contributing to male reproductive dysfunctions often reveal OS conditions and ROS over-production [64,65].

As a class of anti-fungal chemicals, triazole and imidazole derivatives are widely used in multiple fields. Thus, humans are exposed to azoles through environmental and occupational matrices. To date, azoles have been demonstrated to negative affect the development of reproductive organs, resulting in the compromising of male fertility. 

Notably, in a previous study, TEB- and ECO-induced early ΔΨm loss on TM4 SCs [50] suggested the possibility that OS could operate as the *primum movens* in triggering the drug cytotoxicity, especially in view of the strong relationship between ΔΨm and mitochondrial and redox homeostasis [66]. 

The major findings obtained in the present study are consistent with this hypothesis, also confirming the feed-back loop between OS and inflammation. These conclusions are based on the following results: the azole compounds TEB and ECO, alone or in their synergistic combination, induced a significant and sustained increase in ROS generation; these changes were followed by an imbalance in the antioxidant enzyme pattern activities and alterations in the cellular redox state (increase in cellular GSSG content and GSH/GSSG imbalance); this condition occurs in parallel with an increased production of TNF-α and COX-2 expression levels; and finally ROS inhibition rescued SCs from the azole-induced cytotoxic phenotype.

These results were in agreement with previous reports. In particular, OS induced by different classes of antifungal agents, including prochloraz (PCZ), miconazole (MCZ), and ketoconazole (KCZ), is linked to growth inhibitory effects, G0/G1 cell cycle arrest, mitochondrial dysfunctions, and apoptosis [33]. 

In line with this, Ham and colleagues [55] have recently demonstrated that folpet, a phthalimide type of agricultural fungicide used to control crop diseases, induces mitochondrial depolarization and intracellular calcium upregulation with ER stress in TM4 SCs. Additionally, folpet-induced OS mediates antiproliferative effects and apoptotic cell death. Additionally, porcine pre-pubertal SCs exposed to subtoxic doses of nickel oxide nanoparticles (NiO NPs) showed a marked increase in intracellular ROS, DNA damage, activation of caspase-3, and a clear pro-inflammatory stress that resulted in an up-regulation of TNF-α and IL-6 [56]. Endocrine disruptors such as bisphenol A (BPA) induce OS, which affects sperm functions [65]. In addition, the exposure of TM4 SCs to different polycyclic aromatic hydrocarbons (PAHs) results in OS and mitochondrial dysfunction leading to BTB disruption and apoptosis [57]. Potassium dichromate and magnesium sulphate induce ROS generation, an imbalance in the antioxidant enzyme pattern activities (CAT, SOD, and GPx), and DNA damage in testicular cells, including SCs, leading to adverse reproductive abnormalities [67]. In SCs, exposure to the xenoestrogen 4-nonylphenol (NP) promotes ROS generation, ΔΨm loss, decreased cell viability, apoptosis, and necrosis [59,60]. Moreover, monobutyl-phthalate (MBP) can induce OS and apoptosis in human SCs [61].

Here, we showed that TEB-, ECO- and MIX-induced cytotoxicity are associated with increasing ROS accumulation, with TEB showing the weakest activity, while MIX appears to induce the major effect. The prevention of this effect by the ROS scavenger NAC, in addition to confirming the complex interconnection between ROS levels and apoptosis, highlighted an azoles-induced ROS-dependent mechanism of cell death. SCs have evolved several strategies to minimize ROS damage and counteract OS. In this regard, the major ROS-scavenging/modulating enzymes include SOD, which is responsible for the dismutation of the superoxide anion (O_2_^−^) into H_2_O_2_, followed by a set of enzymes such as CAT and GPx operating in concert to remove H_2_O_2_. In addition to the ROS scavenging enzymes, GR and GST are involved in maintaining redox homeostasis either by catalyzing the reduction of GSSG to GSH, or through the conjugation of GSH to xenobiotic substrates for activating their detoxification. Our results show that azole-induced OS may be a consequence of a marked deregulation of the antioxidant defense system. Indeed, a considerable impairment in SOD-specific activity that results in a significant decrease in the SOD/(GPx + CAT) ratio highlights a low ability for O_2_^−^ scavenging. In addition to rapidly reacting with other reactive species to form hydroxyl radicals, O_2_^−^ also promotes iron release from [4Fe-4S] clusters/storage proteins, causing OS, as well as nuclear and mitochondrial DNA damage, ultimately leading to cell injury [68,69]. In a condition of lower SOD activity, the increased levels in CAT activity observed in TEB and MIX may be due to an adaptive mechanism operating to buffer H_2_O_2_ release from cytochromes’ P450 uncoupling reactions [70]. Furthermore, all azole treatments have detrimental effects on GST activity, suggesting a defective detoxification ability. 

As a non-enzymatic antioxidant, the GSH system acts in coordination with the antioxidant enzymatic defense to maintain an intracellular ROS steady-state. The impairment of cellular GSH redox homeostasis caused by GSH oxidation has been reported to contribute to apoptosis [71]. Consistent with these findings, azoles-induced ROS generation and apoptosis occurred in parallel, with a marked and significant increase in GSSG levels, associated with negative and significant variations in the GSH/GSSG ratio, not resulting from changes in intracellular GSH levels. The absence of a GSH pool drop, in our context, would be primarily related to a reduced efficiency of GST and GPx specific activities, two important GSH-utilizing enzymes, rather than to a positive variation in GR activity. This is also evidenced by increases in the GR/(GPx + GST) ratio that reflects the GSH recycling efficiency. In this context, however, we cannot exclude the possibility that de novo GSH synthesis and/or extracellular GSH uptake may also occur. Of note, distinct ROS production, SOD impairment, GSH system status, and significant GR/(GPx + GST) ratio, overall, may explain the lower OS scenario in the TEB condition. 

From a functional perspective, azole-induced alterations in SCs’ redox status may also affect the physiological refill of GSH and SOD provided to spermatogenic cells by their direct interaction. Indeed, SCs cooperate with and support germ cells (which have a limited antioxidant defense system and DNA repair mechanism), in maintaining cellular redox homeostasis [72]. In addition, beyond its role as an intracellular redox buffer, GSH acts as a cysteine donor, essential for protamine biosynthesis in spermatogenic cells [73,74]. 

Several studies have revealed the impact of COX-2 in the regulation of testicular function and male fertility [43,44,45]. Specifically, the pivotal role played by the TNF-α and COX-2/ prostaglandin (PG) system in SCs as a local modulator of testicular activity and a regulatory factor for spermatogenic efficiency in both physiological and pathological conditions has been shown [42,43,75,76]. Interestingly, azoles upregulate the production of TNF-α and induce COX-2 expression, revealing an additional ROS-dependent effect associated with azole-induced cytotoxicity. These findings also suggest further negative effects related to OS, as previously described [33]. Indeed, in this case, NAC can mitigate azole-induced inflammatory effects. These results are in line with Liu and colleagues [77], who reported the ability of nonylphenol (NP) to increase COX-2 expression via ROS-dependent pathways in TM4 SCs. 

Given the centrality of COX-2-mediated PG synthesis in modulating specific key processes, such as the glucose uptake in SCs and steroidogenesis in Leydig cells, azoles may have a negative impact on the spermatogenesis process [43]. Moreover, TNF-α is critical in reducing the steady-state level of occludin, which results in the disruption of SCs’ barrier/BTB function [78]. In in vitro and in vivo conditions, infected SCs with Mumps virus exhibit increased TNF-α production associated with the disruption of the tight junction integrity of the BTB, while TNF-α deficiency prevents BTB dysfunction and spermatids loss [39]. The interaction between SARS-CoV-2 and angiotensin-converting enzyme 2 (ACE2) receptor (expressed on the surface of SCs and Leydig cells) may trigger inflammatory responses leading to Sertoli and Leydig cells dysfunctions [79]. Therefore, the SARS-CoV-2-induced up-regulation of pro-inflammatory cytokines, including TNF-α, is associated with a down-regulation of junctional proteins (e.g., occludin, claudin-11, connexin-43), along with reduced numbers of SCs and the impairment of spermatogenesis [80,81,82]. In contrast to other cytokines, TNF-α is also directly responsible for variations in sperm functions. Specifically, TNF-α abolishes sperm motility, impairs mitochondrial activity, and induces increased levels of DNA fragmentation and sperm apoptosis [83,84].

Although SCs represent a reliable in vitro model to test cytotoxicity in male reproductive systems, our study may have potential limitations. SCs in an in vitro context may differently metabolize xenobiotics compared to the complex in vivo cellular network. Therefore, to mimic the complexity of testicular function properly, a combination of the in vitro and in vivo experimental strategies may be required. 

## 5. Conclusions

To summarize, our findings reveal ROS-dependent cytotoxicity and inflammatory effects induced by azole fungicides in TM4 SCs, highlighting the potential negative role of OS in toxicants-induced male infertility. Moreover, though our study might not reflect the in vivo scenario, it is possible to speculate that the azole-mediated up-regulation of TNF-α and COX2 may play a critical role in BTB disruption and testicular inflammation associated with idiopathic infertility. As NAC attenuates ROS generation and reduces COX-2 expression and TNF-α production, rescuing SCs from azole-mediated cell death, increasing the antioxidant capacity may be a potential strategy to improve SCs’ functionality in toxicant-induced injury.

In light of the above and considering the worldwide use of azole fungicides in different application areas, the results of this study have increased our knowledge of the implications of environmental toxicant mixture in male reproductive disorders, and may contribute to the delineation of a more complete environmental risk assessment.

## Data Availability

All data supporting the findings during this study are available in this manuscript.
